# PET ATTACHMENT AND RETAINED PHYSICAL FUNCTION IN COMMUNITY RESIDING OLDER ADULT PET OWNERS

**DOI:** 10.1093/geroni/igae098.1173

**Published:** 2024-12-31

**Authors:** Erika Friedmann, Nancy Gee, Eleanor Simonsick, Barbara Resnick, Merve Gurlu, Soyeon Shim

**Affiliations:** University of Maryland Baltimore, Baltimore, Maryland, United States; Virginia Commonwealth University School of Medicine, Richmond, Virginia, United States; National Institute on Aging, Baltimore, Maryland, United States; University of Maryland Baltimore, Baltimore, Maryland, United States; University of Maryland Baltimore, Baltimore, Maryland, United States; University of Maryland Baltimore, Baltimore, Maryland, United States

## Abstract

Pet ownership has been associated with slower deterioration in cardiorespiratory fitness (400-M walk time), gait speed, overall functional performance and physical well-being independent of age and comorbidities in older adults with increasing age. As level of pet attachment, an aspect of social support, may impact physical function, we evaluated this association using data from the Baltimore Longitudinal Study of Aging (BLSA). In this longitudinal observational study of 214 pet-owning, generally healthy community-dwelling adults aged 50-100 years (M=68.0, SD=7.8), pet attachment and physical function were assessed during regularly scheduled visits every 1-4 years over 1-13 years (M=7.5,SD=3.6). Linear mixed models with pet attachment, time, and pet attachment by time interaction controlling for age and comorbidities were used to examine the relationship of pet attachment to deterioration in several physical function outcomes. Higher pet attachment was associated with slower deterioration in usual (p< 0.001) and rapid gait speed (p=0.002) and overall physical performance (p=0.009) not cardiorespiratory fitness or physical well-being. Among dog owners only (N=121) attachment did not moderate deterioration in physical function. Among cat owners (N=100), higher attachment was associated with slower decline in physical well-being (p=0.035). We provide important longitudinal evidence that pet attachment is differentially associated with rates of decline in physical function that older adults experience as they age and that attachment to different types of pets may have different relationships to the decline process. Potential explanations for these differences are proposed.

